# Immunogenomic Characteristics of Cell-Death-Associated Genes with Prognostic Implications in Bladder Cancer

**DOI:** 10.3389/fimmu.2022.909324

**Published:** 2022-07-11

**Authors:** Wenhao Xu, Hai-Jia Tang, Aihetaimujiang Anwaier, Wangrui Liu, Xi Tian, Jiaqi Su, Shiyin Wei, Yuanyuan Qu, Hailiang Zhang, Dingwei Ye

**Affiliations:** ^1^ Department of Urology, Fudan University Shanghai Cancer Center, Shanghai, China; ^2^ Department of Oncology, Shanghai Medical College, Fudan University, Shanghai, China; ^3^ Department of Integrated Medicine, Nanjing University of Chinese Medicine, Nanjing, China; ^4^ Affiliated Hospital of Youjiang Medical University for Nationalities, Baise, China

**Keywords:** bladder cancer, cell death, apoptosis, necrosis, ferroptosis, tumor microenvironment, immunotherapy, prognosis

## Abstract

Bladder cancer is one of the most common genitourinary malignant cancers worldwide. Cell death processes, including apoptosis, ferroptosis, and necrosis, provide novel clinical and immunological insights promoting the management of precision medicine. Therefore, this study aimed to evaluate the transcriptomic profile of signatures in cell death pathways with significant prognostic implications in patients with bladder cancer from multiple independent cohorts (n = 1999). First, genes involved in apoptosis (n = 19), ferroptosis (n = 31), and necrosis (n = 6) were analyzed to evaluate the prognostic implications in bladder cancer. Significant genes were included to establish the cell-death index (CDI) of 36 genes that distinguished patients according to high and low risks. Survival analysis using the Kaplan-Meier curves clustered patients based on overall survival (18.8 vs. 96.7 months; hazard model [HR] = 3.12, *P*<00001). Cox proportional hazard model was significantly associated with a higher risk of mortality using 10 external independent cohorts in patients with CDI^high^ (HR = 1.31, 95% CI: 1.04–1.62). To explore immune parameters associated with CDI, microenvironment cell-population-counter algorithms indicated increased intratumoral heterogeneity and macrophage/monocyte infiltration and CD8^+^ T cells in patients with CDI^high^ group. Besides, the CDI^high^ group showed an increased expression of the following immune checkpoints: CD276, PD-L1, CTLA-4, and T-cell exhaustion signatures. Cytokine expression analysis revealed the highest association of IL-9R, IL-17A, IL-17F, GDF7, and IFNW1 with the high-risk group. In addition, 42 patients with BCa receiving immunotherapies were enrolled from a real-world cohort, and expression patterns of three CDI hub genes (DRD5, SCL2A14, and IGF1) were detected using immunohistochemical staining. Patients with triple-negative staining of tumor tissues had significantly higher tumor-associated macrophage abundance, PD-L1 expression, predicted immunocompromised microenvironment, and prominently progressive progression (HR = 4.316, *P* = 0.0028). In conclusion, this study highlights the immunoevasive tumor microenvironment characterized by the higher tumor-associated macrophage infiltration with the presence of immune checkpoint and T-cell exhaustion genes in patients with BCa at CDI^high^ risk who might suffer progression and be more suitable to benefit from immune checkpoint inhibitors or other immunotherapies.

## Introduction

Bladder cancer (BCa) is one of the most common malignant cancers of the urinary system (1), and the 11^th^ most common cause of tumor-related mortality worldwide. Its incidence is four times higher in men than in women (2). Approximately >200,000 annual deaths are reportedly associated with bladder tumors and the treatment for BCa in the last decades (3, 4). At present, transurethral resection or radical cystectomy is the first choice of treatment, and its effect is also the most significant (5, 6). Moreover, the first-line treatment for advanced or metastatic cancer is mainly cisplatin-based chemotherapy, and immunotherapy is considered the second-line treatment (7). In spite of recent advances in clinical treatment, long-term survival rates remain dismal. Thus, promising predictive models to identify patients at risk remain urgently needed to guide the clinical options of active surveillance and therapeutic strategies for patients with Bca.

The tumor microenvironment (TME) maintains complex homeostasis for tumor cell proliferation and development, and its cellular and acellular components together play a supporting role in tumor growth (8). It plays a vital role in maintaining the balance of the immune response and is expected to provide many biomarkers for predicting the Bca prognosis and immunotherapy efficacy (9, 10). The TME has two major cell-death pathways: natural apoptosis in the traditional sense, that is, programmed cell death, a gene-regulated autonomous death method that inhibits the individual anti-tumor immune response, and necrosis, a typical necrosis-like morphology that can cause a significant inflammatory response, as manifested by a large number of inflammatory cell infiltration and activation. The researchers pointed out that these two different cell-death pathways mainly determine the direction of Bca development (11, 12).

Interestingly, ferroptosis is a cell-death mode caused by an imbalance among the production and degradation of intracellular iron and lipid reactive oxygen species (13). As a result of reduced antioxidant capacity and lipid ROS accumulation, cell oxidative death or ferroptosis occurs (14). Several studies have shown that cell-death-related genes can be used to create predictive models to predict cancer occurrence and development (15, 16). Previous studies also stratified patients with lung carcinoma based on the cell-death signature and explored alterations in the transcriptomic profiles of genes in the cell-death pathways, leading to functional enrichment analysis. They found that patients with high-risk cell-death index (CDI) may be better suited to benefit from immunotherapies (17).

However, the genomic and transcriptomic background, underlying mechanisms, and biomarkers of cell death in TME characteristics and immune microenvironment of BCa remain to be elucidated. Therefore, this study aimed to explore the influence of necrosis, apoptosis, ferroptosis, and CDI on the alteration of the TME and clinical phenotypes of BCa.

## Methods

### Patients and Tissue Samples From Online Databases

The RNA sequencing data of 497 patients with BCa was obtained from The Cancer Genome Atlas (TCGA, https://portal.gdc.cancer.gov) with gene IDs converted to gene symbol matrix using USCS Xena (http://xena.ucsc.edu/) as the training cohort. Besides, phenotypic and clinical data of 1531 BCa samples with transcriptomic data from 10 independent cohorts (GSE5479, n = 404; GSE13507, n = 166; GSE19423, n = 48; GSE19915, n = 144; GSE31684, n = 93; GSE32894, n = 308; GSE48075, n = 142; GSE48276, n = 116; GSE69795, n = 61; and GSE70691, n = 49) from the Gene Expression Omnibus (GEO) database as testing cohorts. A total of 42 patients with BCa from the Department of Urology, Fudan University Shanghai Cancer Centre (FUSCC, Shanghai, China) were eventually and consecutively enrolled in analyses from May 2018 to January 2022 with available pathological reports and electronic medical records as the validation cohort.

### Establishment of CDI Using Transcriptomic Gene Expression Profiles

To identify the prognostic implications of cell-death signatures in BCa, we screened and downloaded the gene lists for apoptosis, necrosis, and ferroptosis from the Molecular Signature Database (MsigDB) and Kyoto Encyclopaedia of Genes and Genomes (KEGG) databases in the Gene Set Enrichment Analysis (GSEA) website (https://www.gsea-msigdb.org). The KEGG dataset of apoptosis (n = 331 genes) and necrosis (n = 185 genes) and the FerrDb database gene list of ferroptosis (n = 276 genes) were downloaded (18).

### Univariate Cox Regression and Machine-Learning Analyses

Using gene expression and clinical follow-up information, univariate Cox regression analysis was utilized on genes in each cell-death pathway, and genes with false discovery rate BH-adjusted p-values of <0.1 were considered significant. Further, LASSO regression analysis dimensionality reduction was performed on univariate Cox results, and a risk scoring model, which mainly relies on the R package ‘glmnet’ (v4.0-2), was created (19). To develop prediction models with increased accuracy, cross-validation was implemented for lambda screening, the model corresponding to lamdba.min selected, the expression matrix of genes involved in the model further extracted, and the risk score of each sample calculated using the following formula:


$${\text{RiskScore}}_{i}=\sum_{j=1}^{n}{\text{exp}}_{ji}\times {\text{coef}}_{j}$$

where ‘exp’ represents the expression of the corresponding genes; ‘coef’, the regression coefficient of the corresponding gene in the LASSO regression result; ‘RiskScore’, the expression of genes significantly associated with each sample multiplied by ‘coef’ of the corresponding gene and then summed; ‘i’, sample; ‘j’, genes.

### Survival Analyses

Based on the risk score of the sample, patients were clustered into high- and low-risk groups based on the median cut-off value, and the log-rank test was used to compare the survival curve differences. Then, based on the sample risk score, the ‘survivalROC’ R package was utilized to interpret the time-dependent receiver operating characteristic (ROC) curve and calculate the area under the curve (AUC) value to assess the predictive efficacy model. The ‘meta’ R package was used to perform a meta-analysis of the Cox regression hazard ratio (HR) for all independently validated datasets. The model prediction performance of CDI was compared with BCa prognostic signature models of published studies, including 7-IRG.Sig (20), EMT.Sig (21), TIPS.Sig (22), 8-IRlncRNA.Sig (23), 13-mRNA immune.Sig (24), 14- lncRNA.Sig (25), IRLS.Sig (26), and 6-IRG.Sig (27).

### Evaluation of Differential Parameters of TME, Cytokines, and T Cell Exhaustion Genes in BCa

Based on TCGA transcriptomic data matrix, CIBERSORT, ESTIMATE, MCPcounter, single-sample gene set enrichment analysis (ssGSEA), TIMER, and other algorithms were simultaneously utilized to calculate tumor heterogeneity score, stromal cell score, infiltration levels of tumor-associated fibroblasts, and a range of tumor-infiltrated lymphocyte (TIL) fractions in samples (28–30). Heatmaps were used to show differences in immune infiltration levels between high- and low-risk groups for each risk model, and statistical differences were calculated using the Wilcoxon rank-sum test. To assess activity of anti-tumor immune response in patients with BCa, we evaluated the expression of cytokines and T cell exhaustion genes between differential cell-death groups. Cytokine gene information was downloaded using keyword, “KEGG cytokine-cytokine receptor interaction” (n = 265) (https://www.gsea-msigdb.org). The T cell exhaustion markers were included for investigation as previous reports (8).

### Analysis of Clinical and Pathological Characteristics

Clinical and pathological characteristics include platinum chemosensitivity/resistance, BRCA mut/wild type, homologous recombination deficiency (HRD) mutation, HRD score [the sum scores of the loss of heterozygosity (LOH), telomeric-allelic imbalance (TAI), and large-scale state transitions (LST); median value as cut-off] (31), TCGA molecular type, luminal and basal types (32), immunophenotype (33), lymphoid infiltration status, clinical stage, pathological grade, T stage, gender, age, and smoking history. The Wilcoxon or Kruskal rank-sum test was used to calculate significance levels.

### Differential Gene Identification and Functional Enrichment Analysis

The R package ‘edgeR’ was used to obtain differentially expressed genes between high- and low-risk groups, with significance thresholds of |logFC| > 1 and FDR < 0.01. The R package ‘clusterProfiler’ was used to perform functional enrichment of GO and KEGG databases and screened significant results with a q-value of <0.01 threshold.

### Immunohistochemistry (IHC) Staining Analysis

IHC analysis was implemented to assess the DRD5 (ab32620, Abcam, Cambridge, U.K.), SLC2A14 (QC354Hu01, QchengBio, Shanghai, China), IGF1 (ab263903, Abcam), CD11c (ab52632, Abcam), and PD-L1 (ab205921, Abcam) expression levels following manufacturers’ protocols and previously described procedures (34, 35). The BCa samples were scored according to the degree of cell staining intensity and density.

### Statistical Analysis

The Wilcoxon rank-sum test was implemented to compare the differences between the two groups. The Kaplan-Meier method was utilized to perform survival analysis, and the cut-off value was defined using the median value or ‘survminer’ R package. Log-rank test was used to detect significance. A *P*-value of less than 0.05 was considered statistically significant.

## Results

### Identification of Cell-Death Pathway Factors Associated With Prognostic Values

**Figure 1:**
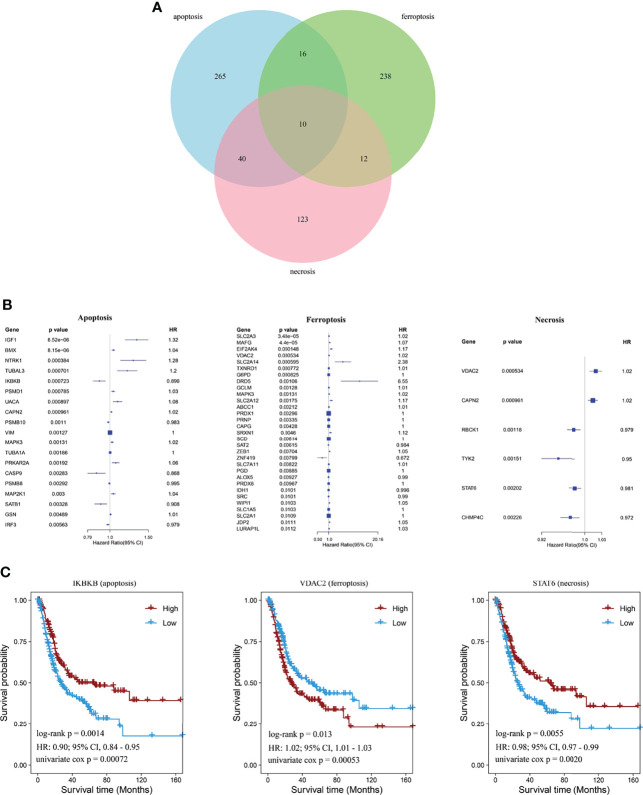
Identification of cell-death pathway factors associated with prognostic values. **(A)** Venn diagram showing unique and identical genes of apoptosis, necrosis, and ferroptosis. **(B)** Forest plot showing significant signatures using univariate Cox regression analysis. **(C)** Kaplan–Meier curves showing prognostic values of *IKBKB, STAT6*, and *VDAC2* of BCa.

Genes expressed in three cell-death pathways (apoptosis, 331 genes; ferroptosis, 276 genes; and necrosis, 185 genes) were identified first, and Venn diagrams were used to screen prognostic indicators in these pathways (**Figure 1A**). Then, a total of 56 prognostic factors were identified, including 19 for apoptosis, 31 for ferroptosis, and six for necrosis, with *IGF1* and BMX as the most significant in apoptosis, *SLC2A3* and *MAFG* in ferroptosis, and *VDAC2* and *CAPN2* in necrosis (**Figure 1B**). For the top prognostic genes in each pathway, patients were divided into two groups based on the median gene expression value to compare survival curve differences (**Figure 1C**). We found that high *IKBKB* expression in apoptosis has a better prognosis, and high *STAT6* expression in necrosis also implies a better prognosis, whereas low *VDAC2* expression in ferroptosis indicates poorer survival of patients.

### Development and Survival Analysis of Patients With BCa Using CDI

**Figure 2:**
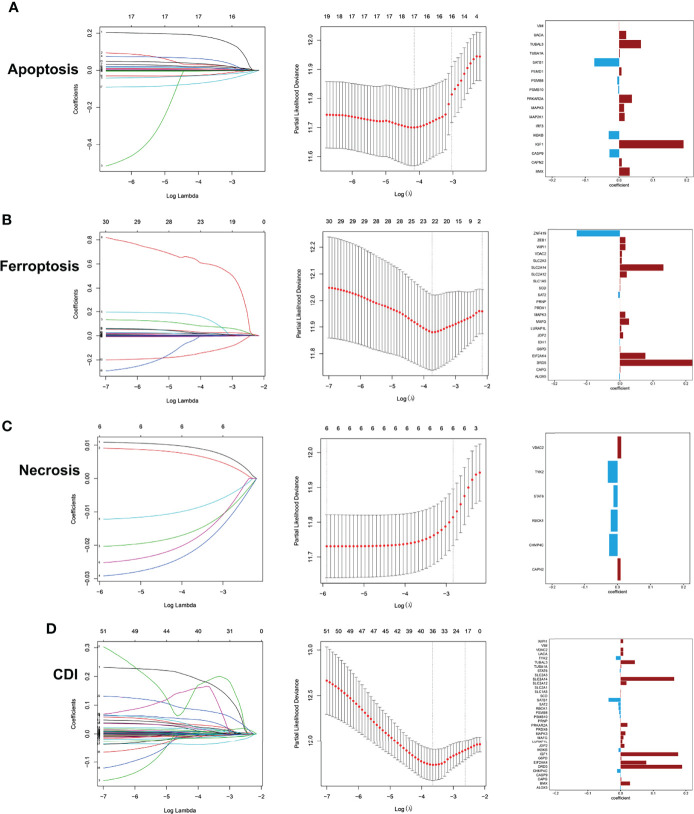
Development of prediction models using LASSO regression analysis with profiles from apoptosis **(A)**, ferroptosis **(B)**, necrosis **(C)**, and CDI **(D)**.

Then, LASSO regression analysis was performed to remove collinearity between factors for dimensionality reduction and construct the prognostic risk model for three cell-death pathways and CDI. The selected genes of each model and their coefficient to the models are illustrated in **Figures 2A–D**. In the CDI pathway, a prognostic model was developed based on 36 genes, with *DRD5*, *IGF1*, and *SLC2A14* expression levels being the most significantly involved in the model classification.

**Figure 3:**
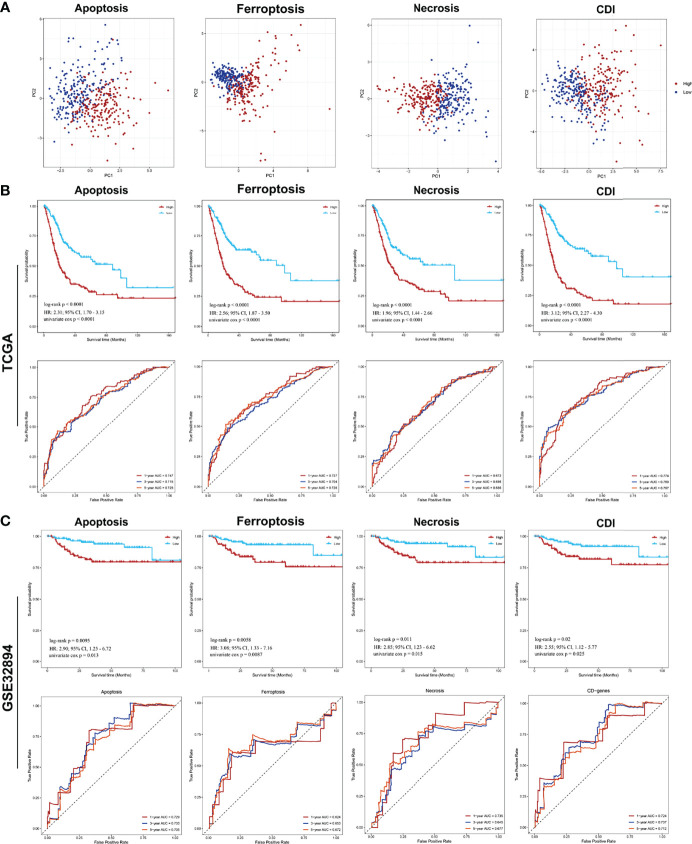
PCA analysis of samples from each pathway and analysis of prognostic model efficacy PCA and survival analysis of cell-death pathways for patients with BCa. **(A)** PCA analysis of the high- and low-risk groups. **(B)** The Kaplan–Meier survival and ROC analysis of high- and low-risk groups for patients from TCGA training cohort and GSE32894 testing cohort.

Next, the principal component analysis (PCA) clearly distinguished the high- and low-risk groups based on CDI and each cell-death pathway (**Figure 3A**). Comparing the two groups of samples in the TCGA database, the low expression group in each cell-death pathway showed markedly better prognosis, with multivariate Cox regression analysis being performed [apoptosis: HR = 2.97, 95% confidence interval (CI), 2.32–3.81; ferroptosis: HR = 3.42, 95%CI, 2.48–4.72; necrosis: HR = 2.41, 95%CI, 1.73–3.34; and CDI: HR = 3.79, 95%CI, 2.92–4.93; *P*<0.0001]. Further, the prognostic power of each prognostic model was evaluated, and the time-dependent ROC curve was plotted. The findings revealed that the 1-, 3-, and 5-year AUC of apoptosis, ferroptosis, and CD genes were all >0.7 and that of necrosis was >0.65, indicating the accuracy of the model and confirming the reliability of the above results (**Figure 3B**). Furthermore, an independent cohort GSE32894 (n = 308) was obtained to further validate the risk score and prognosis difference of each pathway. The prognosis of patients in the low-expression group was higher than that in the high-expression group; however, the difference was not significant. However, the 1-, 3-, and 5-year AUC were all >0.65, indicating a good performance of the risk model (apoptosis: HR = 3.52, 95%CI, 1.46, 8.48; ferroptosis: HR = 3.10, 95%CI, 1.33, 7.22; necrosis: HR = 2.80, 95%CI, 1.20, 6.52; and CDI: HR=2.86, 95%CI, 1.24, 6.62) (**Figure 3C**).

### Assessment of the Relationship Between Clinicopathological Characteristics and CDI

**Figure 4:**
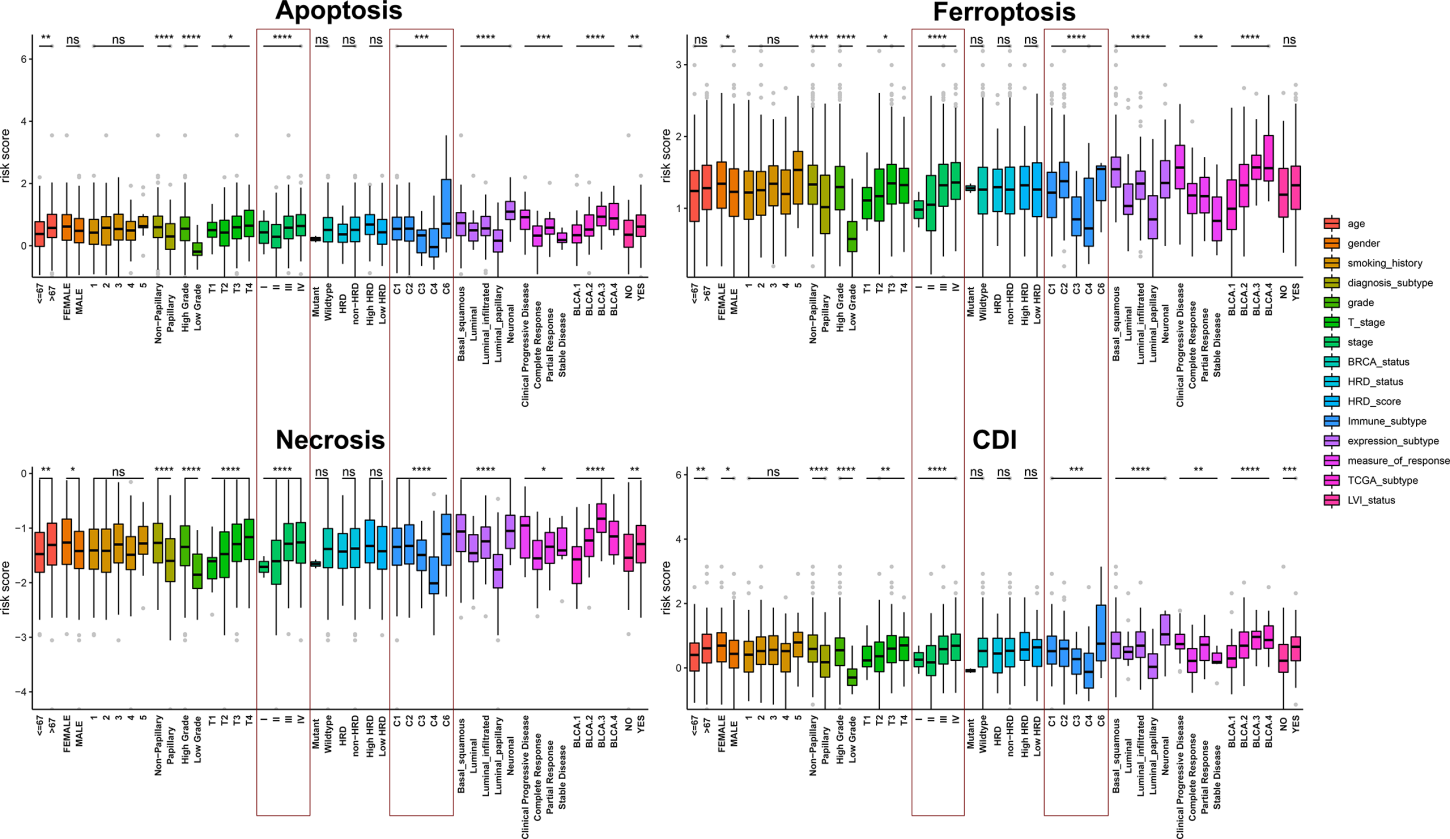
Assessment of the relationship between clinicopathological characteristics and cell-death pathways. *, *P* < 0.05; **, *P* < 0.01; ***, *P* < 0.001; ****, *P* < 0.0001.

By comparing risk scores under different clinical characteristics, all cell-death pathways, diagnostic type, pathological grade, clinical TNM stage, immune subtypes, expression subtypes, and molecular typing were significantly correlated with the cell-death risk score. Specifically, the risk score of the model significantly differs in elevated clinical stages and immune subtypes. Moreover, in all pathways except the ferroptosis pathway, older patients had a significantly higher risk score, and patients with lymphatic invasion had a markedly increased risk score. Additionally, women also had remarkably elevated risk scores compared to men in ferroptosis, necrosis, and CDI. Besides, higher risk scores were associated with less sensitivity to platinum-based chemotherapy. These findings suggested that cell-death models, especially the CDI, was significantly associated with the clinicopathological and molecular subtypes of patients with BCa (**Figure 4**).

### Survival Meta-Analysis of Cell-Death Models

**Figure 5:**
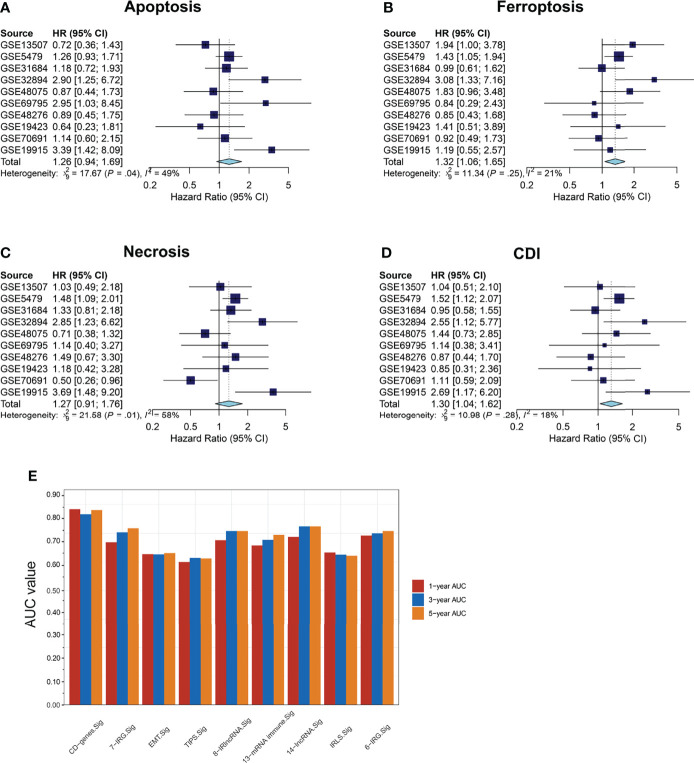
Survival meta-analysis of cell-death pathways. **(A–D)** Meta-analysis of the predictive values of apoptosis, necrosis, ferroptosis, and CDI for prognosis in 1531 BCa samples with transcriptomic data from 10 independent cohorts. **(E)** AUC values of patients at 1, 3, and 5 years were represented by a histogram.

We then collected 1531 BCa samples with transcriptomic data from 10 independent cohorts (GSE5479, n = 404; GSE13507, n = 166; GSE19423, n = 48; GSE19915, n = 144; GSE31684, n = 93; GSE32894, n = 308; GSE48075, n = 142; GSE48276, n = 116; GSE69795, n = 61; and GSE70691, n = 49) from the GEO database for validation. Univariate Cox regression analyses and meta-analysis on the risk score of each model and a meta-analysis on the HR and 95% CI of each risk score were performed. The results revealed that ferroptosis and CDI were significantly associated with survival (ferroptosis: pooled HR = 1.32, 95%CI, 1.06–1.65; CDI: pooled HR = 1.30, 95%CI, 1.04–1.62) (**Figures 5A–D**). Furthermore, compared with the related risk model, the AUC of the patients’ 1-, 3-, and 5-year survival rate in CDI was found to reach approximately 0.80 and exceeded several existing prediction models, indicating that the CDI-based risk model has stronger predictive capacity and may be a promising signature for predicting the prognosis (**Figure 5E**).

### Functional Enrichment Analyses

**Figure 6:**
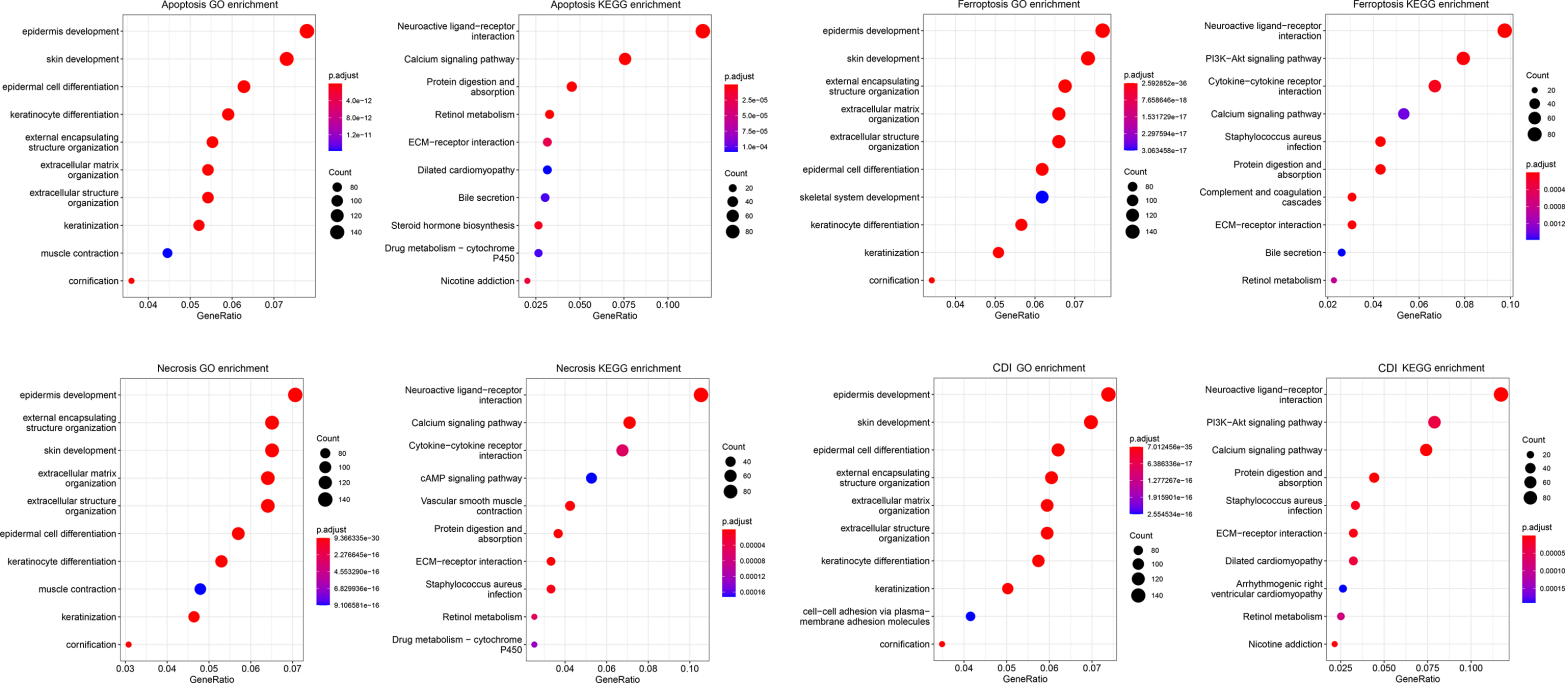
Functional enrichment analysis of cell-death pathways. The bubble chart shows GO and KEGG functional enrichment analyses of cell-death-related genes for each pathway.

Further, functional enrichment analysis was performed in the high- and low-risk groups of each pathway with the data from GO and KEGG databases (**Figure 6**). The three cell-death pathways are respectively mainly enriched in epidermis development in the GO database and neuroactive ligand-receptor interaction in the KEGG database. Apoptosis is mainly enriched in the epidermal development pathway in the GO database, and in the neuroactive ligand-receptor interaction pathway in the KEGG database. Among them, apoptosis was also significantly enriched in the skin development and epidermal cell differentiation in the GO and KEGG databases; necrosis was significantly enriched in external encapsulating structure organization, extracellular matrix organization, and skin development in GO and calcium signaling pathway in KEGG; and ferroptosis was mostly enriched in the skin development in the GO database and PI3K-Akt signaling pathway in the KEGG database.

### Differential TME Parameters Associated With Cell-Death Pathways

**Figure 7:**
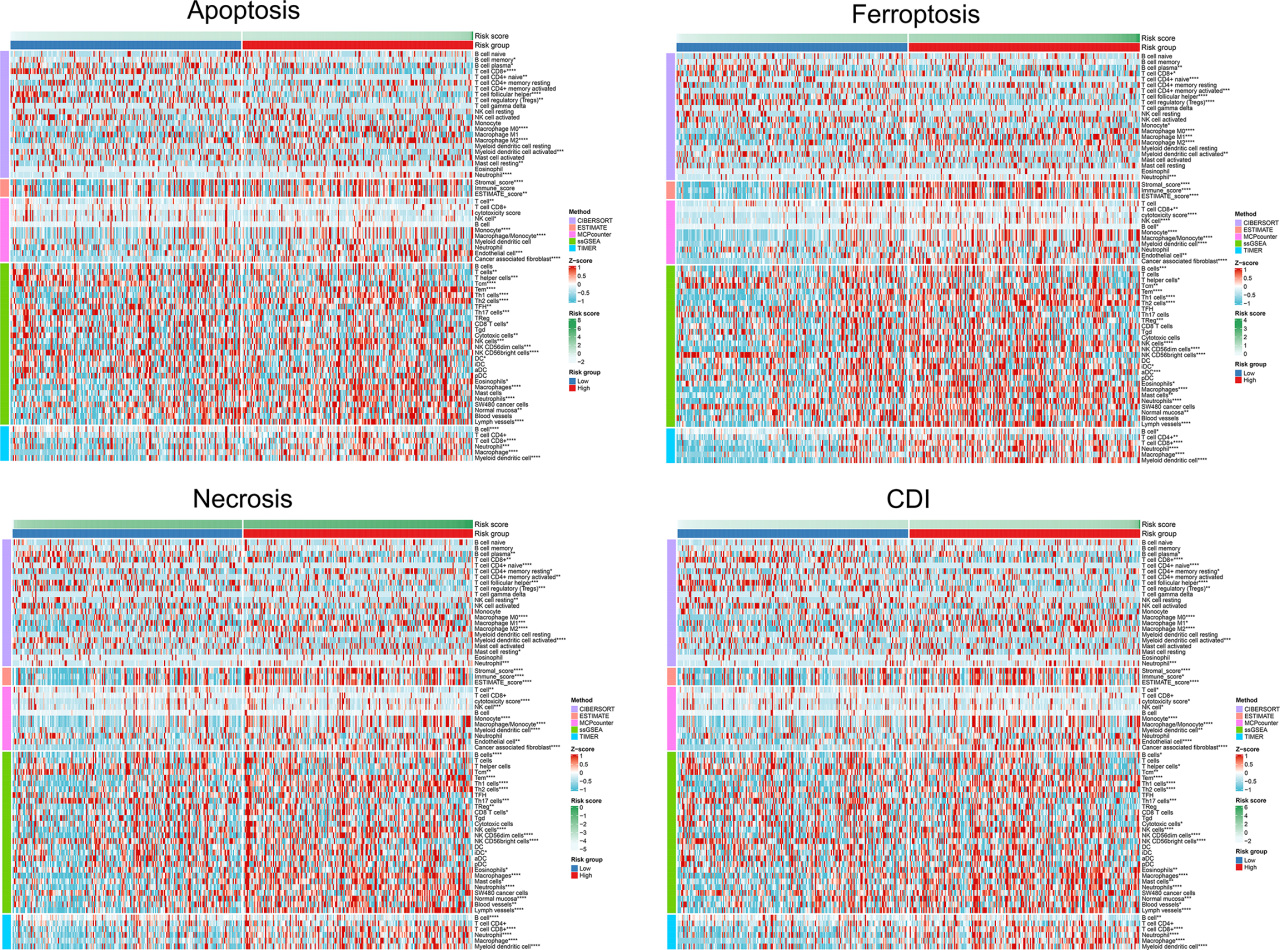
Differential TME parameters associated with cell-death pathways. The clustering heat map showed lymphocytes and other TME parameters for each cell-death risk group. *P < 0.05; **P < 0.01; ***P < 0.001; ****P < 0.0001.

We found that immune infiltration was significantly correlated with the risk scores of each model. Significant associations with specific immune cells were detected in both high- and low-risk groups with different cell-death pathways, suggesting that cell-death pathways might influence patient outcomes through functional insights of immune responses to tumorigenesis. Comparing the immune cell infiltration levels between groups, microenvironment cell-population-counter algorithms indicated that the former group had significantly higher levels of macrophages M0, macrophages M2, neutrophils, cancer-associated fibroblasts, endothelial cells, monocytes, NK cells, eosinophils, lymph vessels, NK CD56dim cells, normal mucosa, Tem, Th1 cells, Th2 cells, and T-cell CD8^+^, whereas the latter group had significantly higher expression levels of B-cell plasma, myeloid dendritic cell activated, T-cell CD4+ naïve, T-cell follicular helper, T-cell regulatory (Tregs), NK CD56bright cells, T cells, and B cells (**Figure 7**). Afterward, we performed prognostic value of lymphocyte-derived and myeloid-derived immune cell infiltration using Kaplan-Meier method, as shown in **Supplementary Figure S1**. Cox proportional hazard model suggested significant prognostic value of macrophages (*P*<0.001) and B cells (*P*=0.028) for BCa patients from the TCGA database. Kaplan-Meier method indicated that elevated CD8 T cells (*P*=0.006) predicted poor prognosis for BCa patients.

### Cytokine Gene Expression, Immune Suppression, and T-Cell Exhaustion Markers

**Figure 8:**
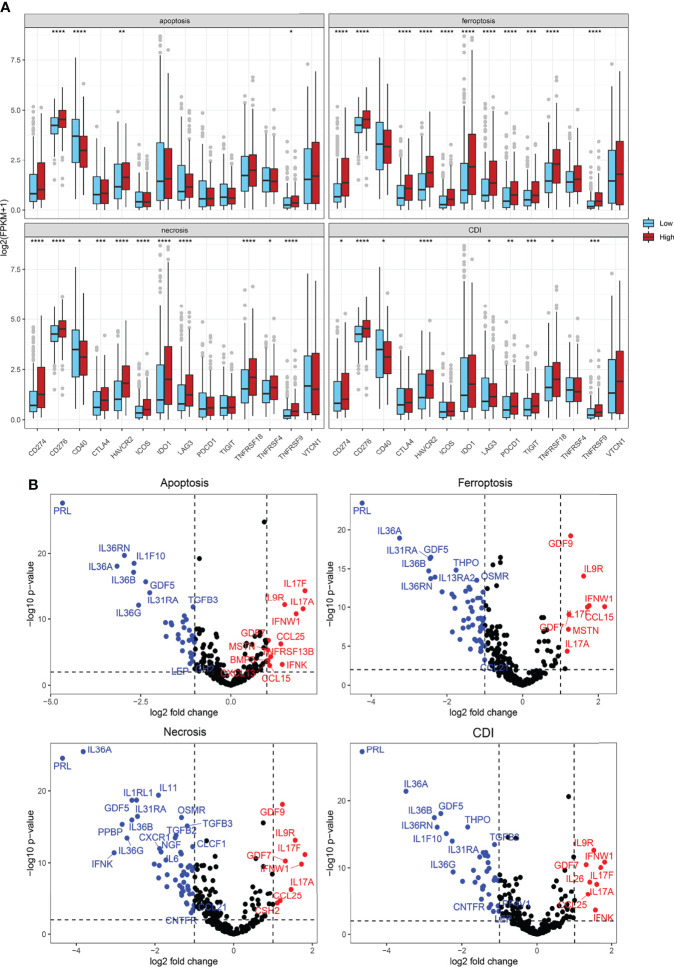
Cytokine, immune suppression, and T-cell exhaustion markers. **(A)** Comparison of immune checkpoint and T-cell exhaustion marker expression levels using Student’s t-test. **(B)** Volcano plot showing significantly expressed cytokines using the “Limma” R package. *P < 0.05; **P < 0.01; ***P < 0.001; ****P < 0.0001.

To explore the mechanism and efficacy of cell-death pathways on anti-tumor immune responses, expression levels of immune checkpoints were detected. The expression level of CD274 and CD276 was significantly elevated in the high-risk of apoptosis, ferroptosis, necrosis, and CDI groups (**Figure 8A**). CTLA4 expression was significantly different in ferroptosis and necrosis clusters. We also found higher expression levels of T-cell exhaustion genes (HAVCR2, TIGIT, PDCD1, IDO1, TNFRSF9, and TNFRSF18) in patients in the high-risk of ferroptosis, necrosis, and CDI groups. Overall, the findings indicated a higher level of immune checkpoints expression and T-cell exhaustion signatures in patients in the high-risk groups of cell-death processes.

Furthermore, differences in cytokine expression were also explored based on the risk score (**Figure 8B**). In the apoptosis group, IL17F, IL9R, IL17A, IFNW1, GDF7, CCL25, MSTN, TNFRSF13B, BMP3, IFNK, CXCL13, and CCL15 expressions were significantly upregulated in the high-risk group, whereas IL36RN, IL36A, IL1F10, IL36B, IL36G, GDF5, IL31RA, and TGFB3 expressions, among others, were significantly downregulated. In the CDI group, IL9R, IFNW1, GDF7, IL17F, IL17A, IL26, CCL25, and IFNK expressions were prominently increased in patients from high-risk groups, whereas PRL, IL36RN, IL36A, IL1F10, IL36B, IL36G, GDF5, and THPO expressions, among others, were significantly downregulated (**Figure 8B**).

### Prognostic and Immunological Implications of CDI in a Real-World Validation Cohort

**Figure 9:**
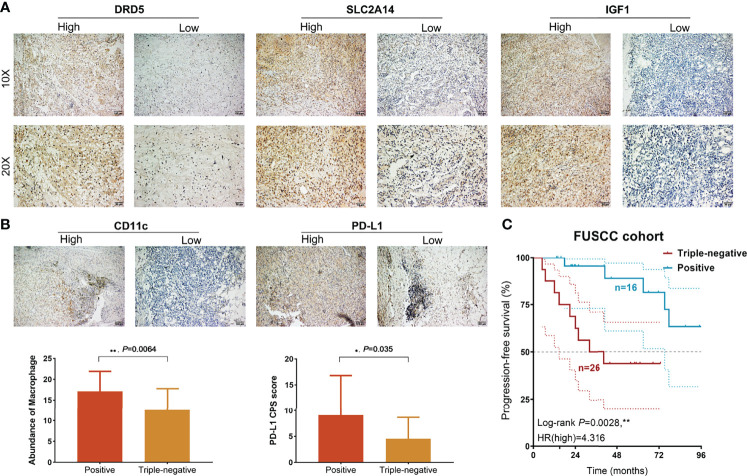
Prognostic and immunological implications of CDI in a real-world validation cohort. **(A)** Immunohistochemical analysis showed DRD5, SLC2A14, and IGF1 expression levels in BCa samples. **(B)** Staining of CD163 and PD-L1 expression levels and comparison of macrophage TAMs abundance and PD-L1 CPS score using unpaired t-test. **(C)** Kaplan–Meier **s**urvival analysis of positive and triple-negative staining groups for patients with BCa treated with immunotherapies (n = 42). **P < 0.01.

To validate the predictive efficacy of CDI in real-world clinical practice, 42 patients with BCa receiving immunotherapies from FUSCC, an Asian real-world cohort, were enrolled, and expression patterns of the three most significant CDI hub genes (DRD5, SCL2A14, and IGF1) were detected using immunohistochemical staining analysis (**Figure 9A**). Besides, we performed correlation analysis between CDI hub genes and immune cells infiltration levels, as shown in **Supplementary Figure S2**. SLC2A14 and IGF1 showed prominently positive association with B cells, CD8^+^ T cells, M1, M2 macrophages, and Tregs, while association between DRD5 expression and immune cell abundance was not significant.

Then, low expression of the three hub genes was identified as a triple-negative group, whereas other samples with neither positive expression were identified as the positive group. Then, CD11c and PD-L1 expressions were detected in BCa tissues, and the abundance of tumor-associated macrophage (TAM) infiltration and PD-L1 CPS score was quantified. These results suggested that TAM infiltration and PD-L1 CPS score were significantly higher than those in the triple-negative group (**Figure 9B**). Next, we found that patients with triple-negative staining of tumor tissues not only showed an immunocompromised microenvironment but also predicted prominent progression in 42 patients with BCa (HR = 4.316, *P* = 0.0028; **Figure 9C**).

## Discussion

Bladder carcinoma exhibits high recurrence rates and notable morbidity and mortality. As precision medicine evolves, accurate disease diagnosis, selection of the most optimal therapeutic strategy for individuals, and early detection and prevention of diseases will all become possible. The integration of high-throughput multi-omics sequencing and bioinformatics integrated analysis technology has led to an era of precision medicine for the diagnosis and treatment of BCa. Immune checkpoint molecule inhibitors have opened the possibility of immunotherapy for BCa, especially for patients with muscle-invasive and metastatic BCa. Besides, the clinical practice of PD-1/PD-L1-related immunotherapies implies potential immunogenicity and has become a novel promising direction for the clinical management of BCa. However, it is necessary to screen out accurate biomarkers to reveal the underlying biological processes of tumor development and assess the effectiveness of immunotherapy in BCa.

In this study, the transcriptomic profile of signatures involved in cell-death pathways with significant prognostic implications was investigated in approximately 2000 patients with BCa. First, significant genes involved in apoptosis (n = 19), ferroptosis (n = 31), and necrosis (n = 6) were analyzed to evaluate their prognostic implications in BCa. The hub genes were included to establish the CDI of 36 genes using machine-learning algorithms. The survival analysis differed patients with BCa in overall survival (18.8 vs. 96.7 months; HR = 3.12, *P*<00001), and the Cox proportional hazard model was significantly associated with CDI^high^ in patients with a higher risk of mortality using 10 external independent cohorts (n = 1531, HR = 1.31, 95%CI: 1.04–1.62). Remarkably, the model prediction performance of CDI was compared with BCa prognostic signature models of published studies and markedly overweighing the existing models, including 7-IRG.Sig (20), EMT.Sig (21), TIPS.Sig (22), 8-IRlncRNA.Sig (23), 13-mRNA immune.Sig (24), 14- lncRNA.Sig (25), IRLS.Sig (26), and 6-IRG.Sig (27). Therefore, various machine-learning algorithms were adopted to establish an accurate and efficient prognostic predictive model for patients with BCa, and solid validations were carried out based on large-scale patient groups from multiple testing cohorts.

Tumor development is also affected by the TME, which can change the tumor angiogenesis by regulating the TME and releasing cytokines (36). To elucidate the mechanism of CDI to facilitate BCa suppression, the TME and individual heterogeneity should be investigated in phenomics after assessing the clinical utility of targeted RNA and DNA molecular profiling (37, 38). To explore the immune parameters associated with CDI, microenvironment cell-population-counter algorithms, including CIBERSORT, ESTIMATE, MCPcounter, ssGSEA, and TIMER, were performed. The results indicated increased intratumoral heterogeneity and macrophage/monocyte and CD8^+^ T-cell infiltration in the high-risk CDI group. Additionally, the CDI^high^ group suggested an elevated expression of immune checkpoints and T-cell exhaustion signatures. CTLA-4 has a higher affinity than CD28, thereby contributing to the suppression of the T-cell-mediated immune responses (39). Besides, Treg cells constitutively express CTLA-4 that further restrains anti-tumor immunity (40). In BCa, the TAMs and CD8^+^ T-cell infiltration revealed a higher expression of PD-1, reduced proliferation of neoantigens, and immunosuppressive circumstance of TME.

Immunogenic cell death has been identified as a promising target to boost T cell anti-tumor immune response and suppress tumor immune evasion (41). By activating cytotoxic and protective signaling modules on specific populations of cells, radiation and chemoimmunotherapy reshapes the immunological tumor microenvironment (42, 43). In this study, T-cell exhaustion genes and cytokines expression (IL-9R, IL-17A, IL-17F, GDF7, and IFNW1) showed significantly higher expression in the high-risk group. As previously studied, alterations in the active tumor-immune cell crosstalk could result in dysfunction of tumor cell escape, immune editing, and even the anti-tumor response (44). Most tumors arise and develop under strong evolutionary pressures imposed by factors such as nutrition, metabolism, immunity, and therapy. These pressures promote the diversification of the tumor microenvironment, ultimately leading to a degree of inherent tumor heterogeneity that enables disease progression and drug resistance (45). To validate the immunophenotype, 42 patients with BCa receiving immunotherapies were enrolled from the FUSCC cohort, and expression patterns of three CDI hub genes (DRD5, SCL2A14, and IGF1) were detected using IHC staining analysis. Patients with triple-negative staining of tumor tissues showed significantly increased TAMs abundance, PD-L1 expression, and prominent progression (HR = 4.316, *P* = 0.0028). Therefore, inconsistent with the findings in other cancers (17, 46), we first proved that CDI cluster could identify the presence of an immunocompromised microenvironment as indicated by the higher infiltration of immune cells with the presence of checkpoint molecules and T-cell exhaustion genes.

## Conclusion

In summary, this study highlights the immunoevasive tumor microenvironment characterized by the higher tumor-associated macrophages infiltration with the presence of immune checkpoint and T-cell exhaustion signatures in patients with BCa at CDI^high^ risk who might suffer progression and be more suitable to benefit from immune checkpoint inhibitors or other immunotherapies.

## Data Availability Statement

The datasets presented in this study can be found in online repositories. The names of the repository/repositories and accession number(s) can be found in the article/**Supplementary Materials**.

## Ethics Statement

Written informed consent was obtained from the individual(s) for the publication of any potentially identifiable images or data included in this article.

## Author Contributions

Conceptualization: WX, H-JT, AA, and WL. Data curation and formal analysis: WX, H-JT, WL, XT, AA, and JS. Funding acquisition: WX, HZ, and DY. Investigation and methodology: WX, H-JT, AA, XT, and WL. Resources and software: WL, JS, SW, YQ, HZ, and DY. Supervision: YQ, SW, HZ, and DY. Validation and visualization: WX, WL, HZ, XT, and AA. Original draft: WX, H-JT, AA, and WL. Editing: SW, YQ, HZ, and DY. All authors contributed to the article and approved the submitted version.

## Funding

This work is supported by Grants from the National Key Research and Development Project (No.2019YFC1316000), “Eagle” Program of Shanghai Anticancer Association (No. SHCY-JC-2021105), the Natural Science Foundation of Shanghai (No.20ZR1413100) and Shanghai Municipal Health Bureau (No.2020CXJQ03).

## Conflict of Interest

The authors declare that the research was conducted in the absence of any commercial or financial relationships that could be construed as a potential conflict of interest.

## Publisher’s Note

All claims expressed in this article are solely those of the authors and do not necessarily represent those of their affiliated organizations, or those of the publisher, the editors and the reviewers. Any product that may be evaluated in this article, or claim that may be made by its manufacturer, is not guaranteed or endorsed by the publisher.

## Abbreviations

BCa, bladder cancer; CDI, cell-death index; CI, confidence interval; CDI, cell-death index; FUSCC, Fudan University Shanghai Cancer Centre; GSEA, gene set enrichment analysis; GSVA, gene set variation analysis; HR, hazard ratio; ICTs, immune checkpoint therapies; IHC, immunohistochemistry; ROC, receiver operating characteristic curve; TCGA, The Cancer Genome Atlas; TME, tumor microenvironment.
