# Supplementary figures and images for: Immunogenomic Characteristics of Cell-Death-Associated Genes with Prognostic Implications in Bladder Cancer
Source: Front Immunol. 2022 Jul 11;13:909324. doi: 10.3389/fimmu.2022.909324 (PMC9309377; doi:10.3389/fimmu.2022.909324)

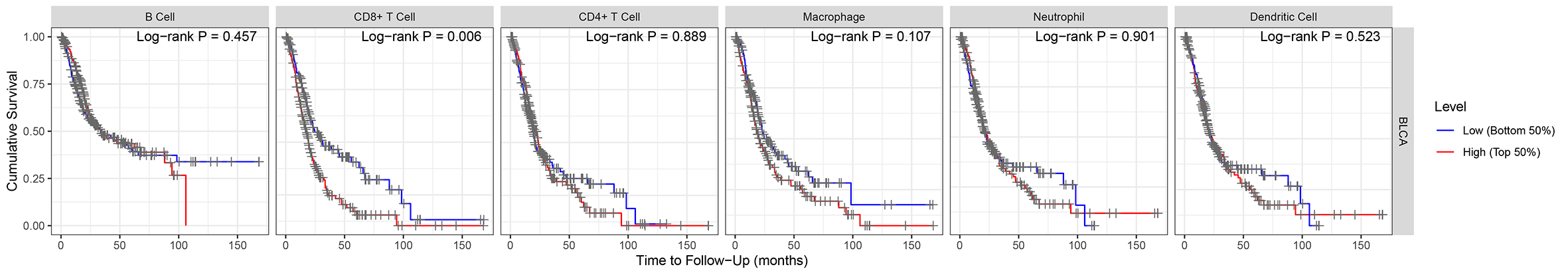

Supplement: Supplementary file 1 [file Image_1.tif]

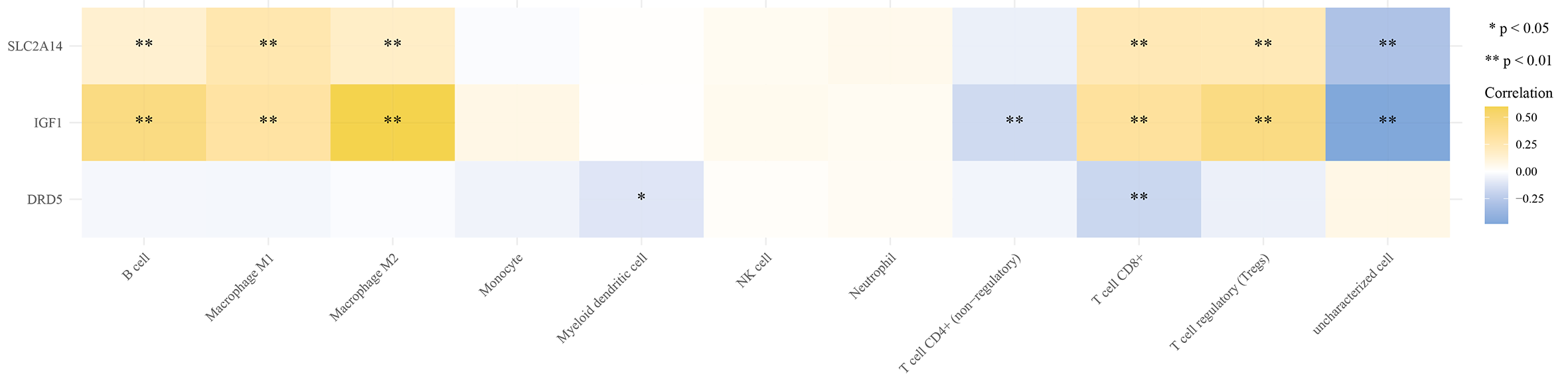

Supplement: Supplementary file 2 [file Image_2.tif]
